# Visualization of the Inferior Alveolar Nerve and Lingual Nerve Using MRI in Oral and Maxillofacial Surgery: A Systematic Review

**DOI:** 10.3390/diagnostics11091657

**Published:** 2021-09-10

**Authors:** Adib Al-Haj Husain, Mark Solomons, Bernd Stadlinger, Rada Pejicic, Sebastian Winklhofer, Marco Piccirelli, Silvio Valdec

**Affiliations:** 1 Clinic of Cranio-Maxillofacial and Oral Surgery, Center of Dental Medicine, University of Zurich, 8032 Zurich, Switzerland; adib.alhaj@gmail.com (A.A.-H.H.); mark.solomons@hotmail.com (M.S.); bernd.stadlinger@zzm.uzh.ch (B.S.); rada.pejicic@zzm.uzh.ch (R.P.); 2 Department of Neuroradiology, Clinical Neuroscience Center, University Hospital of Zurich, University of Zurich, 8091 Zurich, Switzerland; sebastian.winklhofer@usz.ch (S.W.); marco.piccirelli@usz.ch (M.P.); 3 Department of Stomatology, Division of Periodontology, Dental School, University of São Paulo, Butantã 2227, SP, Brazil

**Keywords:** inferior alveolar nerve, lingual nerve, magnetic resonance imaging, oral surgery, anatomy, radiology, systematic review

## Abstract

We evaluate the preoperative visualization of the inferior alveolar nerve (IAN) and lingual nerve (LN) as reported using radiation-free magnetic resonance imaging (MRI). An accurate visualization shall minimize the postoperative risk for nerve injuries in oral and maxillofacial surgery. PubMed MEDLINE, EMBASE, Biosis, and Cochrane databases were selected for the PICOS search strategy by two reviewers using medical subject headings (MeSH) terms. Thirty studies were included in the systematic review. Based on these studies’ findings, the use of black bone MRI sequences, especially 3D short-tau inversion recovery (STIR), provides superior soft-tissue resolution and high sensitivity in detecting pathological changes. Due to the implementation variability regarding scan parameters and the use of different magnetic field strengths, studies with well-designed protocols and a low risk of bias should be conducted to obtain stronger evidence. With improved cost and time efficiency and considering the benefit–risk ratio, MRI is a promising imaging modality that could become part of routine clinical practice in the future.

## 1. Introduction

Accurate diagnostics is an integral part of the clinical routine in today’s pursuit of a multidisciplinary coordinated, personalized, and minimally invasive treatment in high-precision dentistry. In recent decades, advances in biomedical imaging have opened up tremendous possibilities in preoperative diagnostics, specifically in oral and maxillofacial surgery.

In various oral and maxillofacial surgical procedures, such as the removal of impacted mandibular third molars (MTM), orthognathic surgery, or the placement of dental implants, there is a risk of nerve damage to the peripheral branches of the trigeminal nerve, especially to the inferior alveolar nerve (IAN) and lingual nerve (LN). Surgical removal of impacted MTMs represents the most significant risk factor for transient and permanent neurosensory disturbances due to IAN and LN damage, surpassing implant placement and mandibular sagittal split osteotomy [1]. There are heterogeneous data in the literature on the incidence of surgery-related nerve injury, whereby IAN injury is estimated at around 4% (0.4–8.4%) [2,3,4] and LN damage between 0.01 and 2% [5]. Most IAN and LN damages are transient as they recover within 3–6 months. However, the risk of permanent neurosensory disturbances increases if no signs of recovery are observed within this time interval [6]. For affected patients, this permanent nerve damage is accompanied by diminished taste, pronunciation, and general functioning, whereby these psychological and social impairments limit the quality of life [7]. Therefore, accurate preoperative imaging is critical for minimizing the risk of nerve damage and associated unpleasant complications.

Thorough preoperative planning in high-risk oral and maxillofacial surgery includes a risk assessment of adjacent anatomical structures and, consequently, the vulnerable position of the peripheral nerves. The primary diagnostic tool is the commonly used X-ray-based panoramic radiography (PAN), providing a two-dimensional view of the upper and lower jaw, the angulation and position of the MTM, and the surrounding tissues in one single image. In more complex maxillofacial and oral surgical interventions requiring three-dimensional information of the region of interest, cone-beam computed tomography (CBCT) or conventional computed tomography (CT) are indicated. Compared with CT, CBCT generally has lower radiation exposure, which can be further reduced by the use of low-dose protocols and lower costs and is considered the diagnostic gold standard in the oral and maxillofacial region for solid-tissue imaging, although both imaging modalities have deficiencies in soft-tissue information [8]. Despite the insufficient soft-tissue contrast and standardized grayscale value, CBCT is the method of choice for computer-assisted surgery for oral surgeons due to greater accessibility [8]. A significant disadvantage of the increased use in dentistry is the radiation exposure to the thyroid gland [9], especially in the radiation-sensitive group of younger children, due to a possible association with an increased risk of radiation-induced cancer later in life [10,11].

An interesting alternative is radiation-free magnetic resonance imaging (MRI), as it can not only display the osseous boundaries of the mandibular canal such as conventional X-ray-based imaging techniques but also directly depicts the IAN and LN [12]. In recent years, MRI has seen rapid advancements in a wide range of technical improvements and advanced sequences, making it one of the most promising and leading imaging modalities in the head and neck region, offering an increased range of applications in dentistry. The difficulties in visualizing mineralized dental tissue with conventional MRI are due to its dense biological composition, which allows only the limited molecular motion of hydrogen nuclei in water molecules and to the relatively rapid signal decay after radiofrequency excitation, so that the transverse relaxation time (T2) of more than 1 ms required to digitize MRI signals to visualize teeth cannot be achieved [13,14]. Various modifications and further developments such as the use of functional MRI (fMRI), positron emission tomography-magnetic resonance imaging (PET-MRI), diffusion-weighted imaging (DWI), perfusion-weighted imaging (PWI) and the introduction of new sequences such as sweep imaging with Fourier transformation (SWIFT) [15] or “Black Bone” sequences such as 3D-double echo steady state (DESS) and 3D short-tau inversion recovery (STIR) have improved the diagnostic capabilities, allowing the simultaneous visualization of the nervous tissue within the osseous boundaries of the nerve canal [16,17] and the continuous visualization of the extracranial peripheral nerves in general [18], which has a positive impact on various preoperative planning in oral and maxillofacial surgery. Some disadvantages are the limited availability worldwide [19] and higher costs, with motion artifacts, field inhomogeneity, and artifact-inducing dental metallic restorations presenting as the most significant challenges in MR imaging of the oral cavity [20]. With improved cost effectiveness and considering the benefit–risk ratio, MRI presents itself as a promising imaging modality that has the potential to be established in future clinical routines.

Therefore, this systematic review aimed to investigate the following PICO question: does direct visualization of the IAN and LN using MRI provide accurate and feasible preoperative diagnostical information in healthy subjects and patients undergoing oral and maxillofacial surgery?

## 2. Materials and Methods

### 2.1. Search Strategy

This systematic review was conducted according to the preferred reporting items for systematic reviews and meta-analysis (PRISMA) guidelines. The following search strategy was performed to assess the following PICO question: P-population: human studies with healthy subjects or patients older than 12 years undergoing oral and maxillofacial surgery near the IAN and LN; I-intervention: magnetic resonance imaging; C-control: not applicable; O-outcome: feasibility and accuracy of preoperative radiological assessment of the IAN and LN using MRI; and S-study designs: clinical trials. The systematic literature search was performed using medical subject headings (MeSH) terms, precise search terms, and their combinations. The following MeSH terms, search terms, and their combinations were used for the focused PICO question search strategy (Table 1)

### 2.2. Information Sources

The systematic literature search was conducted in the PubMed MEDLINE, EMBASE, Biosis, and Cochrane databases, whereby all English and German publications from 1989 until 1 August 2021 were considered. The search targeted clinical trials performed on human volunteers. The search syntax was classified into population, intervention, comparison, and outcome study design, using MeSH Terms.

### 2.3. Study Selection and Eligibility Criteria

**Figure 1:**
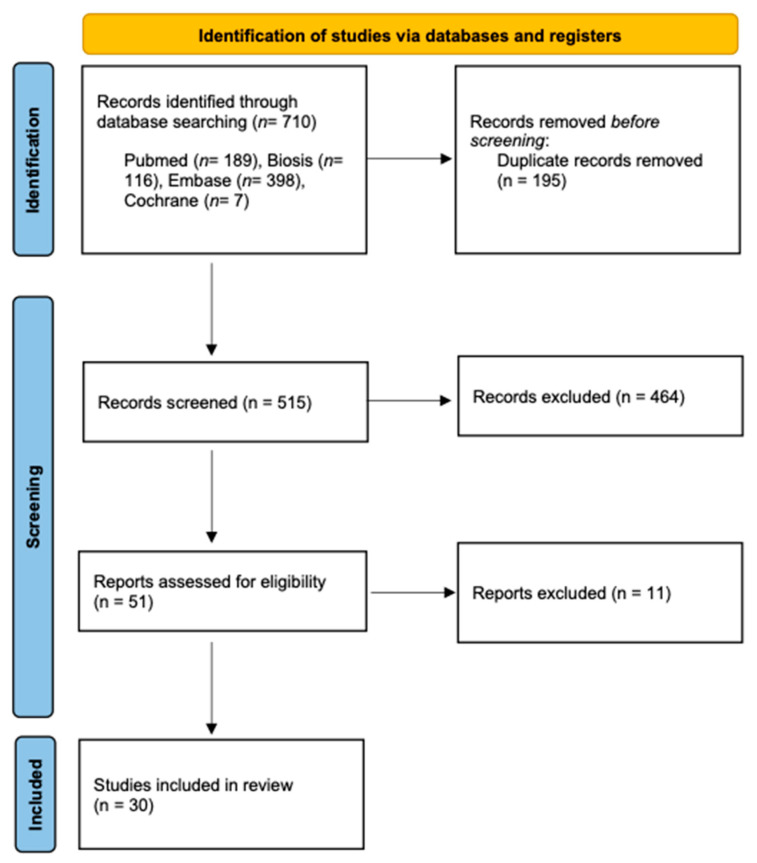
PRISMA flow diagram showing the article selection in this systematic review.

Two reviewers (A.AH. and S.V.) independently conducted the literature searches and study selection to minimize potential reviewer bias. First, the titles and abstracts were screened, followed by the selection of 51 studies to conduct full-text analysis. If any ambiguities appeared, they were resolved through discussion. Finally, 30 studies were selected for the systematic review (Figure 1).

The decision to include studies was based on the following criteria: (1), human studies with healthy subjects or patients undergoing oral and maxillofacial surgery, randomized or nonrandomized controlled trial, cohort studies, and case control studies; (2), study participants aged older than 12 years; (3), all articles refer to the direct visualization of the IAN and LN using MRI; (4), availability of the full text; and (5), publications in English or German language. Articles that fulfilled one of the following criteria were excluded: (1), animal, cadaveric, in vitro studies, narrative reviews, opinion abstracts, letters to the editor, and case reports; and (2), alternative imaging modalities besides MRI.

### 2.4. Data Extraction and Collection

All data were transferred to EndNote (Clarivate, Sydney, Australia) from all databases. First, all duplicates were removed. Second, the data extraction process, screening the titles and abstracts according to the inclusion criteria, and subsequently full-text analysis were performed. Titles and abstracts that did not provide adequate information were also screened by the full texts. If publications did not meet the inclusion criteria, they were excluded. Two reviewers (A.A.H. and S.V.) collected the following data separately: general information (title, author, year, and country), study-specific parameters (study design, number of treated patients, age range of treated patients, and visualized nerves), MRI specific parameters (MRI sequence, field strength, and type of MRI coil), and outcome (feasibility and accuracy).

### 2.5. Risk-of-Bias Assessment and Quality Assessment of Studies

Since MRI is a relatively new scientific field in this area and there are no defined standard MRI sequences or a standard reference test providing direct visualization of the IAN and LN, no index test was performed in most of the included studies, the quality assessment of diagnostic accuracy studies (QUADAS-2) could not be used to assess the risk of bias of the diagnostic test accuracy in reviews based on the following four domains: patient selection, index test, reference standard, and flow and timing [21]. Based on the authors’ experience, the included studies provided an adequate quality; however, some studies lacked accurate statistical methods for sample size calculation, which should always be considered when interpreting results.

## 3. Results

### 3.1. Study Selection

The initial search generated a total of 710 potentially relevant studies, which were scanned for title and abstract, of which 51 articles met the inclusion criteria and underwent full-text screening. Finally, 30 articles were included in the systematic review (Miloro et al., 1997; Nasel et al., 1998; Nasel et al., 1998; Salvolini et al., 2002; Kress et al., 2003; Kress et al., 2003; Kress et al., 2003; Gottschalk et al., 2003; Kress et al., 2004; Mori et al., 2008; Deng et al., 2008; Terumitsu et al., 2008; Krasny et al., 2012; Terumitsu et al., 2013; Cassetta et al., 2014; Cassetta et al., 2014; Assaf et al., 2014; Fujii et al., 2015; Manoliu et al., 2016; Cox et al., 2016; Kotaki et al., 2016; Probst et al., 2017; Terumitsu et al., 2017; Dessouky et al., 2018; Burian et al., 2019; Mazza et al., 2020; Burian et al., 2020; Wu et al., 2020; Beck et al., 2021; Al-Haj Husain et al., 2021) (Table 2). 

### 3.2. Study Characteristics

In the 30 included studies performed between 1997 and 2021, over 1000 patients between 14 and 88 years of age underwent an MRI examination at MR field strengths of 1, 1.5, and 3 Tesla using various MRI sequences (Table 2). Twenty-one studies exclusively investigated the visualization of the IAN, whereby two studies exclusively investigated the visualization of the LN. Seven studies investigated both the IAN and the LN.

## 4. Discussion

This systematic review was conducted to analyze the literature on the visualization of the IAN and LN using MRI. The analysis focused on the influence of MRI sequence selection and field strength on the accuracy and feasibility of MR imaging in the 30 included studies. To the author’s knowledge, no systematic review was conducted on this topic in maxillofacial radiology. Given the advances in MRI technology and the resulting opportunities for preoperative diagnosis, it is useful for discussing the evidence of the individual MRI sequences’ indications and limitations and propose recommendations for the most appropriate application in the decision-making and planning process based on the existing literature. The present review presents a wide range of MRI sequences that illustrate the associated added value and utility of MRI in imaging the IAN and LN depending on the clinical issues.

In oral and maxillofacial surgery, the mandibular canal is routinely visualized using conventional imaging techniques that show only the radiolucent mandibular canal with its surrounding osseous boundaries [22,23]. Apart from the fact that PAN and CBCT cannot directly visualize nervous tissue and are frequently used in MTM surgery, they are associated with an additional increase in radiation-induced cancer incidence by 0.46 owing to CBCT use before MTM surgery [24], which represents one of its main disadvantages, especially when repeated frequently in younger patients [9,10]. For these reasons, and although CBCT is considered the gold standard in dentomaxillofacial imaging for hard tissue, MRI presents a valid attractive option either as a primary diagnostic tool or an imaging modality in follow-ups.

The studies in this systematic review examining the visualization of physiological IAN or LN course in healthy subjects without any associated pathology revealed the possibility of an accurate visualization using a variety of MRI sequences and field strengths. Older MRI studies, conducted around the 2000s, used similar measurement methods that investigated the general visualization of the soft tissues within the mandibular canal [25,26,27]. These three studies used a field strength of 1.0 Tesla and conventional MRI sequences (GE sequence with fat suppression, conventional T1- and T2-weighted sequence, 3D-T1-TFE, and 3D-T2-TFE), demonstrating the visualization of the osseous boundaries and the neurovascular bundle. The neurovascular bundle was displayed as a moderately hyperintense signal, whereas the surrounding osseous boundaries had a lower signal, resulting in good contrast. Limitations were the low field strength of 1 Tesla and the associated low signal-to-noise ratio, which could partly explain the inability to distinguish nerve tissue from blood vessels. Consequently, recent studies have been carried out using a 3 Tesla magnetic field, whereby selected sequences such as 3D FIESTA, 3D SPGR, 3D DESS, and 3D STIR have been investigated for accurate morphological mapping of the IAN and LN [16,17,18,28,29,30]. Cassetta et al. demonstrated the feasibility of the FIESTA and SPGR protocols for accurate visualization of the trigeminal nerve and its branches [29], with reports showing the best results by using the 3D DESS and 3D STIR sequences [16,17], whereby the STIR sequence achieved the most promising results [17]. Simultaneous visualization of the nervous tissues within the osseous boundaries was accurately achieved in all cases, with one report documenting the possibility of determining the precise intraosseous position preoperatively [16]. Burian et al. reconfirmed the excellent feasibility of direct visualization of the complete course of the IAN and the proximal course of the LN. The STIR sequence demonstrated the most promising signal-to-noise ratio and nerve–muscle contrast to noise ratio for the IAN and the LN compared to the previously mentioned sequences. Additionally, the applied MRI sequences allowed excellent soft-tissue contrast and differentiation of the tissue composition of the neurovascular bundle in the mandibular canal in healthy volunteers [17]. Diffusion tensor imaging (DTI), an alternative technique to assess parameters such as diffusivity and anisotropy of microscopic water movement within nerve fibers while obtaining a three-dimensional representation of the IAN, has confirmed these observations by showing better results at higher magnetic field strength and application of black bone MRI sequences such as STIR compared with previous DTI studies [31,32]. Since the signal-to-noise ratio, defined as the ratio between the desired signal intensity and the standard deviation of the unwanted background noise and the image resolution are two of the most important factors describing the quality and accuracy of an imaging technique, the improvement of these parameters should be further pursued in dental MRI technology [33].

To obtain high-resolution images, a high signal-to-noise ratio should be aimed for, which can be achieved by modifying specific scan parameters. Increasing the signal in order to achieve a better performance can be realized by using zero echo time or ultra-short echo time methods, increasing the field of view, time to repeat, or slice thickness, and decreasing the noise by using surface coils, reducing the bandwidth, or increasing the number of excitations [33,34]. However, this is accompanied by a decrease in image resolution, which can be compensated by reducing the field of view and the associated reduction of the voxel sizes by using dedicated mandibular intraoral coils [33,35,36]. In challenging maxillofacial surgical procedures, the acquisition of high-resolution images to detect anatomical variations should be emphasized, as clinically significant information can be of great importance to the surgeon performing the procedure and lead to a better outcome for the patient.

Besides demonstrating the anatomic morphologic course of the IAN, the results of this systematic review show the usefulness of MRI in detecting pathologic neuronal processes after MTM surgery, fractures, or other pathologies. For the diagnosis of peripheral trigeminal neuropathy in the IAN and LN after MTM surgery, MR neurography (MRN) using the water-selective 3D-PSIF sequence with its excellent fat and vascular signal suppression seems to be suitable, as it allows for excellent visualization of various neuropathies such as inflammation, neuroma, nerve transection, or other injuries at a field strength of 1.5 and 3 T [37,38]. However, also in this context, a magnetic field strength of 3 T could display the nerves and estimate the IAN and LN diameter more precisely than 1.5 T due to the higher image resolution. In this regard, the correlation of these MR neurography findings with the intraoperative findings was from good to excellent [38]. Furthermore, using the T1-FFE or FIESTA sequences, other studies measured higher signal intensities distal to the lesions due to higher blood flow in pathologically altered IAN after MTM surgery, which provided information that could be used to predict the duration of postoperative nerve regeneration [28,39]. The same findings could be validated in cases with mandibular fractures, as the neurovascular bundle demonstrated similar different relative signal intensities distal to the lesion after contrast administration compared with healthy patients [40,41]. From this, various experimental setups concluded that MRI imaging could provide information on the pathophysiological processes in the IAN based on signal differences between healthy and injured nerves. In addition, 3DAC-PROPELLER MRI successfully delineated the IAN and LN after traumatic injury and showed a significant correlation between the morphology and the severity of the patient’s sensory disturbance [42]. Burian and colleagues further demonstrated by the application of 3D T1-FFE or 3D STIR sequence, that a good visualization of the fracture and the IAN with high spatial resolution was feasible. The STIR sequence was superior since it was particularly suitable for the visualization of edema within the IAN [43].

For medical conditions of higher complexity such as cysts or tumors associated with displacement of the IAN and LN, specific MRI protocols were investigated. Preoperative MRI could depict the continuous course of the neurovascular bundle and fully identify its positional relationship in patients with minimal cystic processes. However, in larger cysts, the nerve was only partially detectable on each sequence, with the lower magnetic field of 1.5 T being a limitation. Nevertheless, the nerve could be visualized in these patients by evaluating both sections of multidirectional MRI [44]. High-resolution images with a high signal-to-noise ratio and excellent contrast of the continuously displayed IAN could be obtained using the 3D-MPRAGE MR sequence before and after administration of gadolinium in patients suffering from dysesthesia with a mandibular cystic lesion or ameloblastoma [45]. Recently, Wu et al. demonstrated that the contrast-enhanced 3D SPACE STIR sequence could better visualize the morphological changes in peripheral cranial nerve branches and associated pathologies, with higher nerve tissue contrast than the conventional magnetic resonance neurography sequence, in particular in head and neck tumors when assessing the perineural tumor spread [46]. This has opened an interesting complementary diagnostic opportunity for the preoperative planning of tumors that tend to be located in close vicinity of the IAN and the LN.

According to the results of this systematic review, different MRI protocols were able to visualize the morphology of the IAN and LN in healthy individuals and patients with a wide range of ages. Despite the great heterogeneity in the literature regarding scan parameters and the use of different magnetic fields strengths, MRI remains currently the only promising imaging technique that allows noninvasive direct visualization of the neurovascular bundle, distinguishing its nerve tissue from the blood vessels. Since there are no established standards for the use of a specific MRI sequence in IAN- and LN-associated pathology, further studies should be conducted, including randomized control trials, examining the usefulness of each MRI protocol in a specific medical condition to provide an evidence-based understanding of its use and further information on how preoperative radiologic assessment might affect clinical outcome, especially with regard to optimized individualized treatment decision making. With improved costs and time efficiency, the use of black bone MRI sequences such as STIR sequence at a field strength of 3 Tesla, without the use of contrast agent in the preoperative evaluation of wisdom tooth extractions and with contrast agent administration in the involvement of more complex pathologies such as head and neck tumors, could be recommended, as they can provide detailed information on accurate IAN and LN visualization in challenging maxillofacial cases.

## 5. Conclusions

Radiation-free high-resolution MRI for peripheral nerve diagnosis has increased in importance in recent decades. With ongoing technical developments and optimization of MR scan sequences, the structures of the head and neck region can now be radiologically displayed and evaluated in greater detail and reliability. Based on these studies’ findings, the application of black bone MRI sequences, in particular 3D STIR, which have recently been increasingly studied and modified, provide additional preoperative diagnostic information, superior soft-tissue resolution, and high sensitivity in the detection of pathological changes. This may consequently reduce the risk of perioperative IAN and LN injury. Further research could evaluate other factors such as artificial intelligence in maxillofacial radiology and intraoperative use of MRI to visualize the IAN and LN to achieve better outcomes for high-risk oral and maxillofacial surgery patients.

## Figures and Tables

**Table 1:** Search strategy according to the focused question (PICO).

Focused Question (PICO)	Does Direct Visualization of the IAN and LN Using MRI Provide Accurate and Feasible Preoperative Diagnostical Information in Healthy Subjects and Patients Undergoing Oral and Maxillofacial Surgery?	
Search strategy	Population	Human studies (patients and/or healthy subjects), aged older than 12 years undergoing oral and maxillofacial surgical interventions near the IAN and LN. #1—((inferior alveolar nerve [MeSH]) OR (lingual nerve [MeSH]) OR (mandibular nerve [MeSH]) OR (trigeminal nerve [MeSH]))
	Intervention	Magnetic resonance imaging. #2—((magnetic resonance imaging [MeSH]) OR (MRI) OR (nuclear magnetic resonance imaging [MeSH]) OR (NMR) OR (diffusion tensor imaging [MeSH]) OR (DTI) OR (ultra-short echo-time [MeSH]) OR (UTE) OR (maxillofacial imaging)) #3—((visualization) OR (neurography))
	Comparison	Not applicable. Conventional preoperative radiological assessment. #4—((computed tomography [MeSH]) OR (cone-beam computed tomography [MeSH]) #5—(panoramic radiography [MeSH])
	Outcome	Feasibility and accuracy of preoperative radiological assessment of the IAN and LN using MRI. #6—((accuracy) OR (feasibility) OR (signal-to-noise-ratio [MeSH]))
	Search combination(s)	(#1) AND (#2 or #3) OR (#6)

**Table 2:** Characteristics of included studies.

Study Number	Author, Year, Country	Study	Sample Size	Age	Study Objectives	Nerves	MRI Sequences	Field Strengths	Type of MRI Coil	Outcome (Feasibility/Accuracy)
1	Miloro et al., 1997, USA	Assessment of the lingual nerve in the third molar region using magnetic resonance imaging	10	21–35 years	Determination of the exact in situ location of the LN in the third molar region using high-resolution magnetic resonance imaging.	LN	Phase encode time reduction acquisition (PETRA) sequence	1.5 T	surface coil	LN visualization in the molar region using the HR-MRI sequence. Assessment of the nerve diameter, the different shapes of the nerve, and the mean vertical and horizontal distances to the lingual crest and the lingual plate of the mandible.
2	Nasel et al., 1998, Austria	Dental MR tomography of the mandible	5	28–55 years	Presentation of a new imaging technique (dental MR tomography).	IAN	T1- and T2 sequences were performed with gradient echo (GE) and spin echo (SE) techniques -3D T1 GE using a turbo field echo (TFE) -3D T2* TFE	1.0 T	Standard neck-quad coil	Visualization of anatomic features on T1 GE images, neurovascular bundle on T2 images, and nerve exit at the mental foramen.
3	Nasel et al., 1998, Austria	Localization of the mandibular neurovascular bundle using dental magnetic resonance imaging	11	32–57 years	Reliability of visualizing the neurovascular bundle of the mandible using 3D MPR-MRI for the jaws.	IAN	GE sequence with a spectral fat suppression pre-impulse	1.0 T	Standard neck-quad coil	Visualization of the mandibular canal and neurovascular bundle.
4	Salvolini et al., 2002, Italy	Magnetic resonance applications in dental implantology: technical notes and preliminary results	10	N/A	Evaluation of the potential use of magnetic resonance imaging in dental implantology, including the identification of the mandibular nerve.	IAN	T2 TSE, T1 SE and T2 GE	1.0 T	Brain coil	Identification of the intraosseous course of the IAN within the mandibular canal.
5	Kress et al., 2003, Germany	Topography of the inferior alveolar nerve in relation to cystic processes of the mandible in dental MRI	13	15–71 years	Determination whether preoperative MRI could depict the continuous course of the neurovascular bundle and its positional relationship to cystic processes of the mandible.	IAN	T2 TSE-axial, Proton density (PD) FFE-sagittal, PD FFE-coronal	1.5 T	surface coil	High-resolution visualization in minimal cystic processes and identifying the neurovascular bundle in the sagittal and coronal sequences.
6	Kress et al., 2003, Germany	High-resolution MR technique allowing visualization of the course of the inferior alveolar nerve along cystic processes	N/A	N/A	Visualization of the entire course of the IAN in the presence of cystic lesions using high-resolution MRI.	IAN	T2 TSE-axial, T1 TSE sequence, a pd GE—sagittal and coronal	N/A	Head coil	Successful display of the IAN throughout its course.
7	Kress et al., 2003, Germany	MR imaging of traumatic lesions of the inferior alveolar nerve in patients with fractures of the mandible	23	18–64 years	Visualization of the neurovascular bundle in patients with mandibular fracture and investigation of post-traumatic changes in IAN signal intensities.	IAN	Object-oriented sagittal view T1 sequences and pd sequences	1.5 T	Surface coil	Feasibility of displaying the disruption of the neurovascular bundle and intraoperative confirmation of these findings.
8	Gottschalk et al., 2003, Germany	Quantitative analysis of contrast enhanced MRI of the inferior alveolar nerve in inflammatory changes of the mandible	30	18–52 years	Quantitative analysis of the IAN’s signal intensity using contrast-enhanced MRI in inflammatory lesions of the mandible.	IAN	T1- fast-field-echo (FFE) GE-proset-sequence	1.5 T	Surface coil	Assessing the MR signal intensity of the neurovascular bundle and mapping of physiological and pathophysiological states of the IAN.
9	Kress et al., 2004, Germany	High-resolution dental magnetic resonance imaging of inferior alveolar nerve responses to the extraction of third molars	71	18–62 years	Assessment of signal intensity measurements in the neurovascular bundle after third molar extraction treatment using a contrast agent.	IAN	T2 TSE-axial, T1 FFE proset fat saturation-sagittal, contrast agent no delay	N/A	Surface coil	Accurate visualization of the neurovascular bundle, with postoperatively increased signal intensity measurements at the defined regions of interest.
10	Mori et al., 2008, Japan	Diffusion tensor tractography for the inferior alveolar nerve (V3): initial experiment	10	21–35 years	Evaluation of diffusion tensor tractography (DTT) in the mandible to visualize the IAN.	IAN	Single-shot echo planar imaging (EPI) sequence with a SENSE factor of 2	1.5 T	SENSE Flex S coil	The automatic fiber tracking method made the IAN visualization possible, providing more information overlapping with T1 images.
11	Deng et al., 2008, China	High-resolution magnetic resonance imaging of the inferior alveolar nerve using 3-dimensional magnetization-prepared rapid gradient-echo sequence at 3.0 T	18	18–42 years	Visualization of the anatomy of the IAN in healthy subjects and patients with mandibular diseases.	IAN	3D magnetization-prepared rapid gradient-echo (3D MP-RAGE)	3.0 T	Standard neck and CP spine array coil	High-resolution images with a high signal-to-noise ratio and excellent contrast of the IAN in the intact mandible and in patients with mandibular trauma could be obtained using the 3D-MPRAGE MR sequence.
12	Terumitsu et al., 2008, Japan	Morphologic evaluation of the inferior alveolar nerve in patients with sensory disorders by high-resolution 3D volume rendering magnetic resonance neurography on a 3.0-T system	16	N/A	Evaluation of the morphological appearance of the IAN in patients who reported persistent sensory dysfunction due to unilateral IAN injury.	IAN	T1 fast spoiled gradient-recalled echo (fast SPGR) sequence with fat suppression (Chemical shift selective pulse (CHESS))	3.0 T	8-channel neurovascular phased-array coil	High-resolution 3-dimensional volume rendering magnetic resonance neurography (3DVR-MRN) visualized the course of the IAN and its morphological changes.
13	Krasny et al., 2012, Germany	Anatomic variations of neural canal structures of the mandible observed by 3-Tesla magnetic resonance imaging	64	N/A	Evaluation of anatomical variations of the mandibular canal, the neurovascular bundle, mental canal, incisive canal, and the nutrient canals in patients having nonspecific head or neck pain.	IAN	modified 3D high-resolution T2 sampling perfection with application optimized contrasts using different flip angle evolution (SPACE) sequence	3.0 T	head and neck coil	Precise visualization of the IAN’s anatomical variations in the fully dentate mandible of the volunteers, providing the detection of entire structures and structural abnormalities accurately.
14	Terumitsu et al., 2013, Japan	Evaluating fine structure of the injured trigeminal nerve and tissue using 3D anisotropy contrast imaging on a 3.0-T system	20	N/A	Distinguishment of the course of the nerve fibers from the connective tissue mass in patients with injured IAN or LN.	IAN + LN	Three-dimensional anisotropy contrast periodically rotated overlapping parallel lines with enhanced reconstruction (3DAC PROPELLER) diffusion-weighted imaging (DWI)	3.0 T	N/A	Successful delineation of the lesion from the surrounding connective tissues, providing textural details indicating possible severity of sensory dysfunction.
15	Cassetta et al., 2014, Italy	High-resolution 3-T MR imaging in the evaluation of the trigeminal nerve course	78	17–71 years	Qualitative evaluation of the course of four segments of the trigeminal nerve, including the extracranial segments of the mandibular nerve.	IAN + LN	3D T2 fast imaging employing steady-state acquisition sequence (3D FIESTA); T1 fast spoiled gradient recalled echo sequence (3D SPGR)	3.0 T	8-channel neurovascular phased-array coil	Accurate visualization of the trigeminal nerve and its branches, especially the mandibular nerve, with high inter- and intra-rater variability.
16	Cassetta et al., 2014, Italy	3.0 Tesla MRI in the early evaluation of inferior alveolar nerve neurological complications after mandibular third molar extraction: a prospective study.	196	19–32 years	Investigation of the use of 3.0 Tesla MRI to predict possible sensitivity disturbances qualitatively and quantitatively in the IAN after third molar extraction treatment.	IAN	3D FIESTA; 3D SPGR	3.0 T	8-channel neurovascular phased-array coil	No differences in the course of the IAN were observed preoperatively and postoperatively with good inter- and intra-rater variability.
17	Assaf et al., 2014, Germany	Evaluation of four different optimized magnetic-resonance-imaging sequences for visualization of dental and maxillo-mandibular structures at 3 T	12	25–63 years	Evaluation of four optimized noncontrast MRI sequences on the visibility of osseous and dental maxillary and mandibular structures on healthy subjects.	IAN + LN	T1; fat saturated T1; fat saturated T2 and constructive interference steady state (CISS).	3.0 T	20-channel head-and-neck coil	The evaluated sequences provided excellent 2D and 3D visualization of osseous and dental structures, allowing its use in the differentiation of the mandibular canal, the IAN, the mental foramen, and the LN.
18	Fujii et al., 2015, Germany	Visualization of the peripheral branches of the mandibular division of the trigeminal nerve on 3D double-echo steady-state with water excitation sequence	86	17–88 years	Detectability of the six extracranial branches of the mandibular nerve.	IAN + LN	3D double-echo steady-state with water excitation sequence (3D-DESS-WE)	3.0 T	20-channel head-neck coil	Successful visualization of the LN and IAN based on a 5-point scale.
19	Manoliu et al., 2016, Switzerland	MR neurographic orthopantomogram: ultrashort Echo-Time Imaging of Mandibular Bone and Teeth Complemented with High-Resolution Morphological and Functional MR Neurography	10	20–50 years	Investigation of a new technique for MR neurographic orthopantomograms that uses ultrashort echo time (UTE) imaging to visualize bones and teeth.	IAN	3D PETRA single-echo sequence; 3D DWI reversed fast imaging with steady-state precession (3D-PSIF); 3D T2 SPACE sequence with short tau inversion recovery (STIR) fat suppression; accelerated diffusion-tensor-imaging (DTI) prototype sequence (2D SMS-accelerated RESOLVE)	3.0 T	64-channel phased array coil	The image quality was excellent for all sequences using the suggested MR neurographic orthopantomogram. All sequences performed excellently in the anatomical delineation of the mandibular canal and IAN with high diagnostic confidence. In particular, the 3D PETRA and 3D PSIF provided a complete representation of the mandibular canal in all volunteers.
20	Cox et al., 2016, USA	Magnetic resonance neurography in the management of peripheral trigeminal neuropathy: experience in a tertiary care centre	17	14–69 years	Applicability of magnetic resonance neurography in the assessment of peripheral trigeminal neuropathies in patients with suspected trigeminal neuropathies (IAN and LN).	IAN + LN	CISS 3D sequence axial, T1 and T2 spectral adiabatic inversion recovery (SPAIR) sequences axial, DTI axial, 3D STIR SPACE coronal, 3D DW PSIF coronal	1.5 T	Multichannel head coil	Excellent image quality could be achieved in the treatment of peripheral trigeminal neuropathy, by additionally showing moderate to excellent correlation with intraoperative results.
21	Kotaki et al., 2016, Japan	Diffusion tensor imaging of the inferior alveolar nerve using 3T MRI: a study for quantitative evaluation and fibre tracking	46	20–36 years	Qualitative and quantitative visualization and fibre tracking of the IAN in healthy participants using diffusion tensor imaging.	IAN	DTI: SE-based single-shot EPI with fat suppression by STIR sequence, 3D T1 with MP-RAGE	3.0 T	16 channel neck and head coil	DTI is a feasible technique for quantitative analysis and imaging of the IAN, providing quantitative data of the diffusivity and anisotropy of microscopic water movement within nerve fibers while simultaneously obtaining three-dimensional visualization of the nerve fibers.
22	Probst et al., 2017, Germany	Magnetic resonance imaging of the inferior alveolar nerve with special regard to metal artifact reduction	7	N/A	Investigation of the capacities and limitations of IAN MRI in patients with IAN disruption with respect to metal artifacts.	IAN	WARP sequences, T2 TSE coronal, T2 TSE axial, T2 TSE Spectral Presaturation with Inversion Recovery (SPIR) parasagittal, T1 TSE parasagittal, T2 TSE parasagittal, 3D CISS triplanar reconstructions, 3D T1 volumetric interpolated breath-hold examination (VIBE) triplanar reconstructions	1.5 T and 3.0 T	12-channel head coil with an additional four-channel surface coil	Assessment of better image quality at 3.0 T. Achievement of average to excellent image quality of the IAN and its structural changes by using the WARP sequence taking into account the severity of metallic artifacts. The findings correlated with clinical symptoms.
23	Terumitsu et al., 2017, Japan	High-contrast high-resolution imaging of post-traumaticmandibular nerve by 3DAC-PROPELLER magneticresonance imaging: correlation with the severity of sensory disturbance	19	27–62 years	Assessment of high-resolution magnetic resonance imaging of the IAN and LN after trauma-related injury and the correlation between the morphological classification and the degree of severity of the sensory disorder and morphologic characteristics of the surrounding connective tissue.	IAN + LN	3DAC PROPELLER DWI pulse sequence	3.0 T	Neurovascular coil or a custom 3-inch surface coil	Successful visualization of the internal structures within the lesion, delineation of the IAN and LN after traumatic injury and display of the morphologic features in relation with the severity of the patient’s sensory disturbance.
24	Dessouky et al., 2018, USA	Role of MR neurography for the diagnosis of peripheral trigeminal nerve injuries in patients with prior molar tooth extraction	42	N/A	Evaluation of the diagnostic accuracy of MR neurography for the assessment of damaged peripheral trigeminal nerves in patients with previous third molar extraction and its correlation with the intraoperative findings.	IAN + LN	3D PSIF, T1, T2 fat suppressed, DWI	1.5 T and 3.0 T	Multichannel head coil	Reliability and accuracy for the diagnosis of post-traumatic nerve injury after molar tooth extractions. Neuropathy could be determined by various qualitative and quantitative criteria.
25	Burian et al., 2019, Germany	MRI of the inferior alveolar nerve and lingual nerve—anatomical variation and morphometric benchmark values of nerve diameters in healthy subjects	30	21–32 years	Examination of the reliability of various black bone MRI sequences for direct visualization of the IAN and LN in healthy volunteers.	IAN + LN	3D STIR 3D DESS, 3D T1 FFE	3.0 T	16-channel Head Neck Spine Coil	Excellent feasibility of direct visualization of the complete course of the IAN and the proximal course of the LN.
26	Mazza et al., 2020, Italy	Appearance of normal MRI anatomy of the lingual nerve using steady-state free precession sequences at 3-T	24	18–84 years	Application of a steady-state free precession (SSFP) MRI sequence to investigate the direct visualization of the LN.	LN	FIESTA	3.0 T	Head and neck multiarray coil	Visualization of the LN throughout its course from the mandibular nerve to the mylohyoid muscle in excellent image quality.
27	Burian et al., 2020, Germany	High resolution MRi for quantitative assessment of inferior alveolar nerve impairment in course of mandible fractures: an imaging feasibility study	30	16–57 years	Visualization of the IAN course in patients with unilateral mandibular fracture and healthy patients using different “black bone” MRI sequences.	IAN	3D STIR, 3D DESS, 3D T1 FFE	3.0 T	16-channel head-neck-spine coil	Black bone MRI sequences generally allowed good visibility, with the 3D T1 FFE sequence and the 3D STIR sequence providing good visualization of the fracture and the IAN.
28	Wu et al., 2020, China	Visualization of the morphology and pathology of the peripheral branches of the cranial nerves using three-dimensional high-resolution high-contrast magnetic resonance neurography	35	18–69 years	Visualization of the peripheral cranial nerves, including the IAN and LN in healthy and pathological volunteers.	IAN	T1 TSE axial, T2 TSE axial, T2 turbo inversion recovery magnitude (T2-TIRM) coronal, conventional (noncontrast enhanced) 3D SPACE STIR (cMRN), contrast enhanced T1-SPAIR and contrast enhanced 3D SPACE STIR (HRHCMRN) axial.	3.0 T	head and neck coil	Very good visualization of the morphology of the IAN and LN and the pathologies affecting them.
29	Beck et al., 2021, Austria	Is MRI a viable alternative to CT/CBCT to identify the course of the inferior alveolar nerve in relation to the roots of the third molars?	53	N/A	Evaluation of the spatial relationship between the IAN and the mandibular third molar (MTM) using MRI or CT/CBCT images.	IAN	PD T2 TSE FS axial, PD TSE FS coronal	3.0 T	64 channel head-and-neck coil	Observation of good inter- and intra-rater concordance in evaluating the spatial relationship between IAN and MTM. In addition, MRI images provided benefits in the identification of accessory IAN in comparison to CT/CBCT.
30	Al-Haj Husain et al., 2021, Switzerland	Mandibular third molar surgery: intraosseous localization of the inferior alveolar nerve using 3D double-echo steady-state MRI (3D-DESS)	19	18–63 years	Evaluation of the IAN’s intraosseous position within the mandibular canal’s osseous boundaries in patients undergoing third molar extraction treatment using preoperative cone-beam computed tomography and magnetic resonance imaging. Assessment of a conversion factor between the two imaging modalities.	IAN	3D-DESS-WE	3.0 T	64 channel head-and-neck coil	Accurate simultaneous visualization of the nerve tissues within osseous boundaries. A conversion factor from IAC in CBCT and MRI to IAN in MRI was determined.

## Data Availability

Not applicable.

## Abbreviations

3DAC PROPELLER	Three-dimensional anisotropy contrast periodically rotated overlapping parallel lines with enhanced reconstruction
CISS	Constructive interference in steady state
DESS-WE	Double-echo steady-state with water excitation sequence
DTI	Diffusion tensor imaging
DWI	Diffusion weighted imaging
EPI	Echo planar imaging
FFE	Fast field echo
FIESTA	Fast Imaging Employing Steady-state Acquisition
GE	Gradient echo
MP-RAGE	Magnetization-prepared rapid gradient-echo
PD	Proton density
PETRA	Phase encode time reduction acquisition
PSIF	Reversed fast imaging with steady-state precession
SE	Spin-echo
SPACE	Sampling perfection with application optimized contrasts using different flip angle evolution
SPAIR	Spectral adiabatic inversion recovery
SPGR	Spoiled gradient recalled
SPIR	Spectral Presaturation with Inversion Recovery
STIR	Short tau inversion recovery
T1	T1 weighted image
T2	T2 weighted image
T2*	T2 relaxation
TFE	Turbo field echo
TIRM	Turbo inversion recovery magnitude
TSE	Turbo spin echo
VIBE	Volumetric interpolated breath-hold examination
